# Transcriptome Sequencing Unveils a Molecular-Stratification-Predicting Prognosis of Sarcoma Associated with Lipid Metabolism

**DOI:** 10.3390/ijms25031643

**Published:** 2024-01-29

**Authors:** Yuheng Hong, Lin Zhang, Weihao Lin, Yannan Yang, Zheng Cao, Xiaoli Feng, Zhentao Yu, Yibo Gao

**Affiliations:** 1 Department of Thoracic Surgery, National Cancer Center/National Clinical Research Center for Cancer/Cancer Hospital, Chinese Academy of Medical Sciences and Peking Union Medical College, Beijing 100021, China; 2 Department of Thoracic Surgery, National Cancer Center/National Clinical Research Center for Cancer/Cancer Hospital & Shenzhen Hospital, Chinese Academy of Medical Sciences and Peking Union Medical College, Shenzhen 518116, China; 3 Department of Pathology, National Cancer Center/National Clinical Research Center for Cancer/Cancer Hospital, Chinese Academy of Medical Sciences and Peking Union Medical College, Beijing 100021, China; 4 Central Laboratory & Shenzhen Key Laboratory of Epigenetics and Precision Medicine for Cancers, National Cancer Center/National Clinical Research Center for Cancer/Cancer Hospital & Shenzhen Hospital, Chinese Academy of Medical Sciences and Peking Union Medical College, Shenzhen 518116, China; 5 Laboratory of Translational Medicine, National Cancer Center/National Clinical Research Center for Cancer/Cancer Hospital, Chinese Academy of Medical Sciences and Peking Union Medical College, Beijing 100021, China; 6 State Key Laboratory of Molecular Oncology, National Cancer Center/National Clinical Research Center for Cancer/Cancer Hospital, Chinese Academy of Medical Sciences and Peking Union Medical College, Beijing 100021, China

**Keywords:** sarcoma, lipid metabolism, SQLE, molecular classification, drug target

## Abstract

Sarcomas are heterogeneous connective tissue malignancies that have been historically categorized into soft tissue and bone cancers. Although multimodal therapies are implemented, many sarcoma subtypes are still difficult to treat. Lipids play vital roles in cellular activities; however, ectopic levels of lipid metabolites have an impact on tumor recurrence, metastasis, and drug resistance. Thus, precision therapies targeting lipid metabolism in sarcoma need to be explored. In this study, we performed a comprehensive analysis of molecular stratification based on lipid metabolism-associated genes (LMAGs) using both public datasets and the data of patients in our cohort and constructed a novel prognostic model consisting of squalene epoxidase (SQLE) and tumor necrosis factor (TNF). We first integrated information on gene expression profile and survival outcomes to divide TCGA sarcoma patients into high- and low-risk subgroups and further revealed the prognosis value of the metabolic signature and immune infiltration of patients in both groups, thus proposing various therapeutic recommendations for sarcoma. We observed that the low-risk sarcoma patients in the TCGA-SARC cohort were characterized by high proportions of immune cells and increased expression of immune checkpoint genes. Subsequently, this lipid metabolic signature was validated in four external independent sarcoma datasets including the CHCAMS cohort. Notably, SQLE, a rate-limiting enzyme in cholesterol biosynthesis, was identified as a potential therapeutic target for sarcoma. Knockdown of SQLE substantially inhibited cell proliferation and colony formation while promoting the apoptosis of sarcoma cells. Terbinafine, an inhibitor of SQLE, displayed similar tumor suppression capacity in vitro. The prognostic predictive model and the potential drug target SQLE might serve as valuable hints for further in-depth biological, diagnostic, and therapeutic exploration of sarcoma.

## 1. Introduction

As a group of large heterogeneous connective tissue malignancies of mesenchymal origin, sarcomas are the second most common solid tumors, accounting for about 20% of cancer-caused deaths in children and adolescents due to their extreme aggressiveness and metastasis [1,2,3]. Based on the anatomical site of occurrence, sarcomas can be historically categorized into two large subclusters, osteosarcomas and soft tissue sarcomas [2], and both subclusters consist of a series of histological subtypes. Recent scientific achievements have revealed that the number of subtypes at the molecular level has increased constantly. Conventional therapeutic strategies, including surgical excision, chemotherapy, immunotherapy, and targeted therapy, have been employed in patients suffering from advanced or metastatic sarcoma [4,5]. However, the prognosis of patients with metastatic sarcoma has hardly progressed in the past thirty years; nearly 30% of patients still succumb to sarcoma, with a five-year survival rate of only 16% despite systemic therapy [6,7].

Alteration of energetics and metabolism in cells is well known as a hallmark of cancer [8]. Emerging evidence has revealed that multiple signaling pathways, proteins, and relevant mechanisms get involved in the alteration of how cells use distinct molecules and metabolites to support abnormal cellular proliferation, dissemination from the primary tumor, the establishment of a secondary tumor, and immune evasion. Lipids, nucleic acids, and proteins are building blocks of biological membranes and essential components that construct cells. In addition, lipids get involved in metabolism and energy storage and play vital roles as molecules for signal transduction for many cellular activities. Lipid metabolism including the uptake, synthesis, and hydrolysis of the lipid is strictly regulated to maintain cellular homeostasis [9,10]. Malignant cells in the tumor microenvironment, in which nutrient availability is consistently altering during tumor progression, utilize lipid metabolism to sustain their rapid proliferation, invasion, and metastasis [11,12].

Preclinical and clinical studies on a battery of inhibitors targeting the enzymes in lipid metabolism have been investigated [9,13]. To target cholesterol synthesis, statin family drugs, such as HMG-CoA reductase (HMGCR) inhibitors, are currently being investigated as anticancer agents in multiple trials [14]. Studies revealed that first-line therapy combined with statins extended the survival of patients with colorectal cancer and metastatic pancreatic cancer [15,16].

Certain sarcoma classifications based on transcriptome profiles have been proposed in recent years, but molecular subclusters based on the consensus clustering analysis of lipid metabolism-associated genes (LMAGs) have not yet been established. To refine the current treatment regimen with more personalized therapeutic options, in this study, we systemically developed an LMAG-based prognostic model, which was confirmed to be an independent risk factor for sarcoma patients. The prognosis value of metabolic signatures, immune infiltration, and the drug sensitivity of the subtypes were explored. Furthermore, SQLE was screened out as a novel drug target for sarcoma patients. In addition, the novel prognostic model and therapeutic target were well verified by four other independent datasets including the CHCAMS sarcoma cohort and in vitro experiments. Our work could help further improve the efficacy of precision medicine for sarcoma.

## 2. Results

### 2.1. Identification of Subclusters Based on LMAGs in Sarcoma

**Figure 1:**
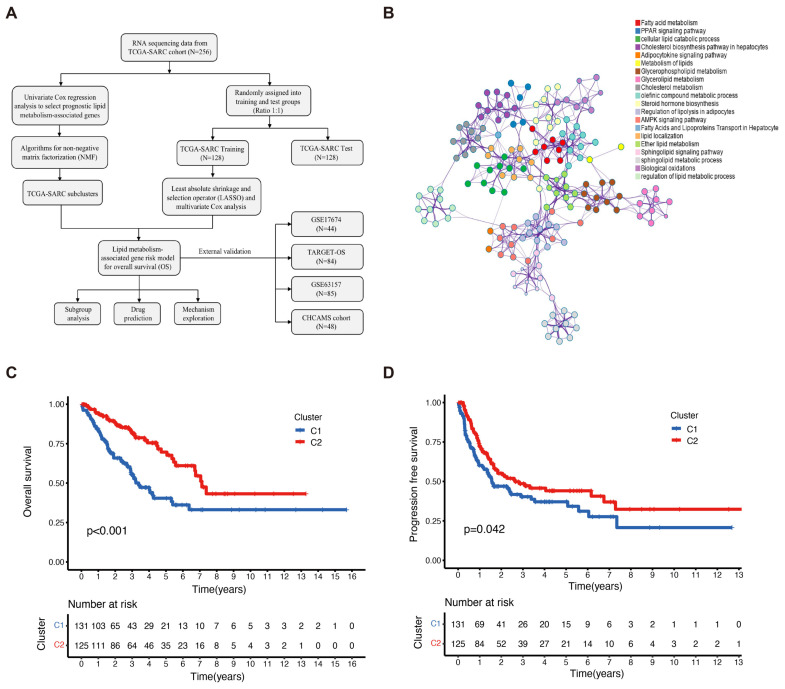
Identification of sarcoma subclusters utilizing unsupervised NMF clustering in the TCGA–SARC cohort. (**A**) A flow chart illustrating the study. (**B**) Metascape showing the annotations of lipid metabolism-associated genes and the biological processes. (**C**) The overall survival (OS) and (**D**) progression-free survival (PFS) of the patients in clusters 1 and 2 of the TCGA–SARC cohort. TCGA, The Cancer Genome Atlas; TCGA–SARC, the whole TCGA sarcoma cohort. CHCAMS, Cancer Hospital, Chinese Academy of Medical Sciences; LMAGs, lipid metabolism–associated genes.

A flow chart was generated to display this study (Figure 1A). Firstly, we performed a Metascape clustering analysis to illustrate the biological functions and the involved signaling pathways of the lipid metabolism-associated genes, as shown in Figure 1B. Lipid metabolism-associated genes were screened by an MAD value > 0.5, a significant prognostic value, *p* < 0.05, and subjected to unsupervised NMF analysis in order to identify gene expression profiles through the decomposition of the original matrix into two non-negative matrices in the TCGA-SARC cohort. The k value was determined by using a comprehensive correlation coefficient. Two subclusters were, thereafter, set according to the optimal k value (k = 2) and the matrix heatmap (Figure S1A). The consensus matrix heatmaps were displayed at k values of 2 to 7 (Figure S1B). The two subclusters were designated as cluster 1 (C1) and cluster 2 (C2). C1 patients showed inferior OS (*p* < 0.001) and progression-free survival (PFS) (*p* = 0.042) compared to those in C2 (Figure 1C,D).

### 2.2. Prognostic Values of LMAGs in Sarcoma

Fifty-five LMAGs were identified to be correlated with OS by using univariate COX regression analysis (Table S2), and the correlations among these genes are demonstrated in Figure S2. Based on the optimal penalty parameter, the LASSO regression analysis determined that the optimal number of LMAGs involved in the model was seven, including ACAA1, DHCR7, PLA2G4C, PTDSS1, PTGER3, SQLE, and TNF (Figure S3A,B). Furthermore, an analysis of the multivariate Cox regression was performed and an LMAG prognostic model consisting of SQLE and TNF was constructed (Figure S3C). The expressions of SQLE and TNF in sarcoma demonstrated the opposite prognostic prediction (Figure S3D,E). Taking the median lipid metabolism-associated risk score as the cut-off value, the sarcoma patients were subsequently divided into high- and low-risk assessment groups (Table S3). TCGA-SARC high-risk group patients showed higher expressions of SQLE and lower TNF; however, both genes in the low-risk group exhibited opposite expressions (Figure S3F,G). The dataset GSE99671 provides RNA-sequencing results of osteosarcoma tissues and normal bone samples [17], with which we analyzed differentially expressed genes and found that SQLE was upregulated (*p* = 0.0087) but TNF was downregulated (*p* > 0.05) in osteosarcoma (Table S4).

**Figure 2:**
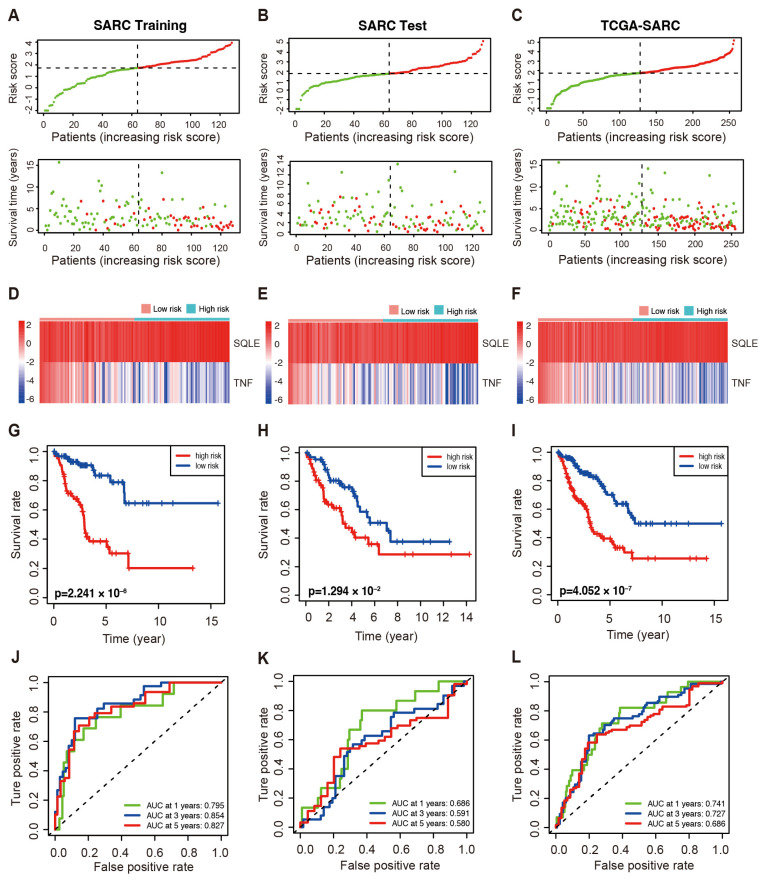
The prognostic value of the LMAGs risk model in the indicated cohorts of TCGA–SARC patients. (**A**–**C**) The distributions of risk score and OS in the training (**A**), test (**B**), and whole cohorts (**C**). Red: High–risk; green: low–risk. Heatmaps demonstrated SQLE and TNF expression in the training (**D**), test (**E**), and whole cohorts (**F**). Kaplan–Meier curve of OS in the high– and low–risk groups in the training (**G**), test (**H**), and whole cohorts (**I**). The ROC analyses in the training (**J**), test (**K**), and whole cohort (**L**).

Patients in the high-risk group of the three cohorts suffered a shorter survival duration as demonstrated by the distribution of risk scores and survival status (Figure 2A–C). The expression profiles of SQLE and TNF are illustrated by heatmaps (Figure 2D–F). In addition, survival analyses of the training set revealed that patients at low risk exhibited significantly longer OS than the high-risk group (*p* < 0.001, Figure 2G). Moreover, both the test cohort and the whole TCGA-SARC cohort showed similar outcomes compared to the training set (Figure 2H,I). The ROC curve of the training cohort consistently showed AUC values of 1-, 3-, and 5-year survival at 0.795, 0.854, and 0.827, respectively. The ROC curves of these cohorts indirectly reflect the robust predictive ability of the prognostic risk score (Figure 2J–L). Subsequently, the efficacy of the prognostic model in the subgroups was evaluated to further verify the predictive value of the model in patients with various clinical features. The patients in the TCGA-SARC cohort were divided into subgroups according to gender (male and female) (Figure S4A,B), age (≥65 and <65) (Figure S4C,D), metastases (metastatic and non-metastatic) (Figure S4E,F), and disease types (UPS, DDL, and LMS) (Figure S4G–I). In all subgroups, especially the UPS group, patients at low risk exhibited significantly longer survival than those at high-risk.

### 2.3. The LMAGs Risk Model as an Independent Prognostic Factor for Sarcoma

**Figure 3:**
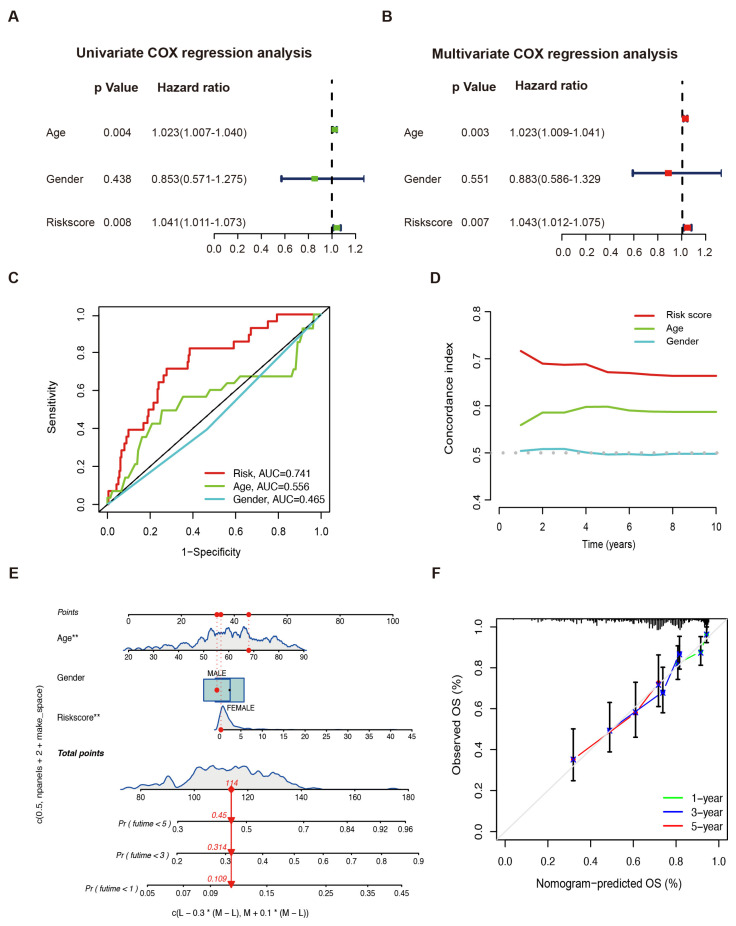
Sarcoma prognosis based on the LMAGs risk model. Risk score and clinical feature analyses based on univariate (**A**) and multivariate (**B**) Cox regressions. (**C**) The ROC curve and (**D**) the C–index of risk score and independent clinical factors. (**E**) The nomograph of risk score and independent clinical factors to predict 1-, 3-, and 5-year OS. * *p* < 0.05, ** *p* < 0.01. (**F**) Calibration curves.

In the univariate Cox regression analysis, risk score and age significantly correlated with the OS (risk score: HR = 1.041, 95% CI: 1.011–1.073, *p* = 0.008; age: HR = 1.023, 95% CI: 1.007–1.040, *p* = 0.004) (Figure 3A). Risk score and age were independent prognostic indicators for the OS as demonstrated by the multivariate Cox regression analysis (risk score: HR = 1.043, 95% CI = 1.012–1.075, *p* = 0.007; age: HR = 1.023, 95% CI: 1.009–1.041, *p* = 0.003, Figure 3B). The predictive ability of the clinical features was inferior to the risk score in the TCGA-SARC cohort (Figure 3C,D). A nomogram integrating the prognostic model, age, and gender was constructed to improve survival prediction. With each item scored according to the actual condition, patients were given a total score to predict the survival rate within 5 years, as shown in Figure 3E. Notably, the calibration curves exhibited that the predicted 1-, 3-, and 5-year OS provided by the nomograph match the actual values, suggesting that the constructed nomograph is accurate (Figure 3F).

### 2.4. Immune Microenvironment and Infiltration in Sarcoma

**Figure 4:**
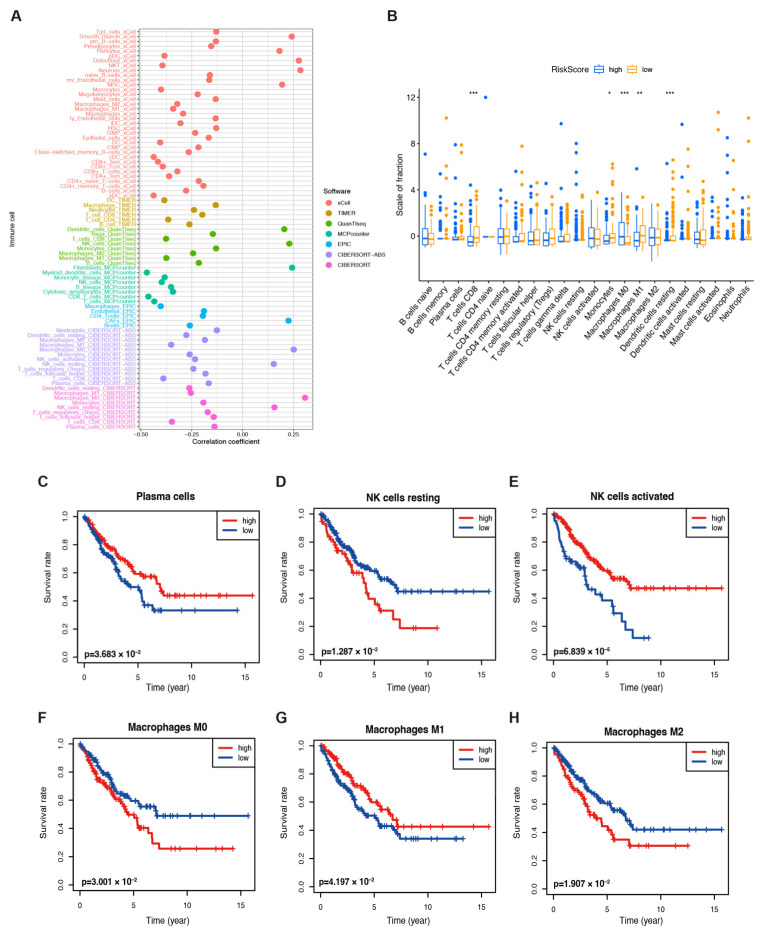
Immune infiltration in TCGA–SARC patients. (**A**) The immune cell bubble of risk groups. (**B**) The abundance of different infiltrating immune cells in the two risk subgroups. * *p* < 0.05, ** *p* < 0.01, and *** *p* < 0.001. The correlation between infiltrated (**C**) plasma cells, (**D**) resting NK cells, (**E**) activated NK cells, (**F**) M0 macrophages, (**G**) M1 macrophages, and (**H**) M2 macrophages and the OS of patients.

To better understand the performance of the model, we first evaluated the correlation between the risk scores and immune infiltration by conducting a Spearman analysis, and the results are shown in Figure 4A and Table S5. We then analyzed the tumor-infiltrating immune cells by using the CIBERSORT tool in high- and low-risk TCGA-SARC samples. The produced boxplot demonstrated that CD8+ T cells, M0 and M1 macrophages, monocytes, and resting dendritic cells were evidently abundant in the low-risk samples (Figure 4B). Additionally, plasma cells, resting and activated NK cells, M0, M1, and M2 macrophages were significantly correlated to OS (Figure 4C–H). According to these findings, an activated immune response might contribute to anti-tumor effects in low-risk groups.

**Figure 5:**
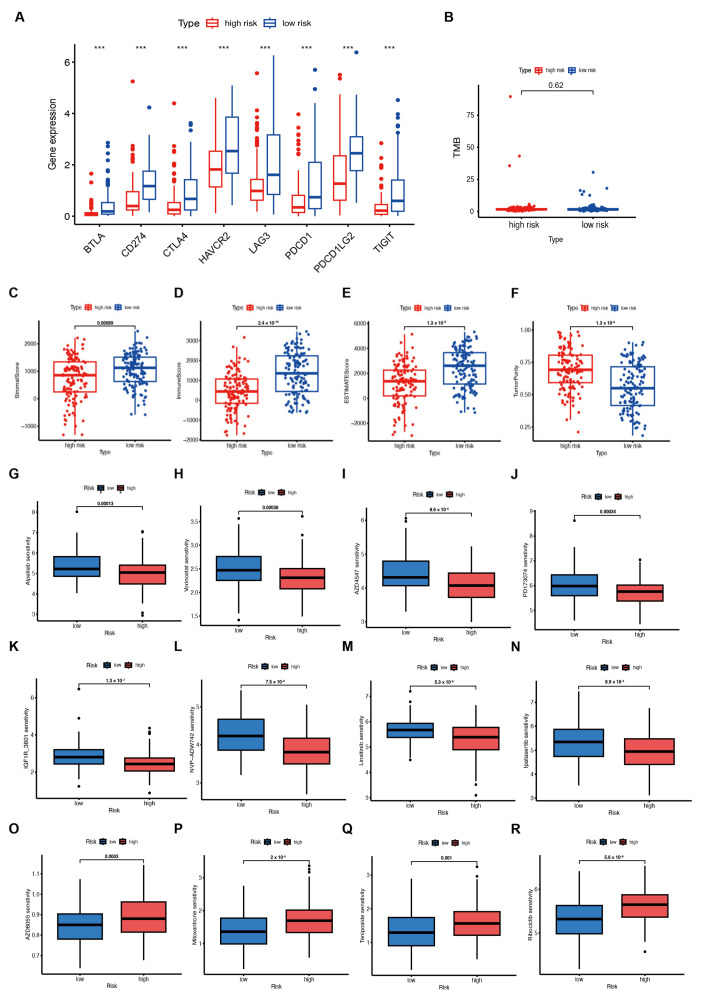
Immunotherapy response and drug sensitivity prediction in patients from the TCGA–SARC cohort. (**A**) Immune checkpoint gene expression in high– and low–risk groups. *** *p* < 0.001. (**B**) Tumor mutational burden (TMB) in both groups. (**C**–**F**) The Estimation of STromal and Immune cells in MAlignant Tumours using Expression data (ESTIMATE) analyses of the TCGA–SARC cohort. (**G**–**R**) Drug sensitivity in the two groups.

Although emerging evidence has revealed that immunotherapy is a treatment for tumors, biomarkers to predict its efficacy are still under investigation. Therefore, the TCGA-SARC dataset was further analyzed to identify whether lipid metabolic signatures are associated with immune checkpoints. Additionally, we investigated the immune checkpoint gene expression and found that BTLA, CTLA4, CD274, HAVCR2, PDL1, LAG3, PDL2, and TIGIT expression were significantly increased in the low-risk group (Figure 5A). This indicates that immunotherapy may be more effective for those at low risk. However, no significant difference was observed in TMB in both groups (Figure 5B). A significant upregulation of these genes in low-risk patients was also found in the TARGET-OS and GSE63157 cohorts (Figure S5A,B). In addition, ESTIMATE was applied to assess the infiltration of immune and stromal cells, and the results showed higher immune, stromal, and ESTIMATE scores (Figure 5C–E). In contrast, tumor purity was lower in low-risk patients than in those at high risk (Figure 5F). The TARGET-OS dataset showed similar results (Figure S5C–F), but no significant difference in the GSE63157 scores was observed (Figure S5G–J). Together, these findings suggest that immunotherapy may be suitable for low-risk patients with an immune-infiltrated microenvironment.

### 2.5. Analysis of Drug Sensitivity

Based on the GDSC database, we examined the association between risk score and drug sensitivity to find possible drugs that might be effective for patients with high risk scores. The results indicated that patients with lower risk scores were more likely to be sensitive to drugs. Finally, we used the GDSC database and the oncopredict R package to estimate the different chemosensitivity of low- and high-risk patients. Patients in the low-risk group showed more sensitivity to a variety of chemotherapeutic agents, including inhibitors of PI3K (Alpelisib), HDAC (Vorinostat), FGFR (AZD4547 and PD173074), and IGF-1R (IGF1R_3801, NVP-ADW742, and Linsitinib), as well as AKT (Ipatasertib) (Figure 5G–N). Patients in the high-risk groups demonstrated better sensitivity to inhibitors of mTOR (AZD8055), topoisomerase II (Mitoxantrone and Teniposide), and CDK4/6 (Ribociclib) (Figure 5O–R).

### 2.6. Validation of the LMAGs Risk Model Using the TARGET and GEO Datasets

We verified the reliability of the LMAG-based risk model with the enrolled TARGET-OS, GSE17674, and GSE63157 datasets, and the risk scores distribution together with the survival status of the three cohorts revealed that patients at high risk have a shorter survival duration (Figure S6A–C). The heat maps of the two risk model genes are shown in Figure S6D–F. Furthermore, the risk model was evaluated by applying a KM survival analysis and the data revealed that patients with low risk showed superior OS (*p* < 0.001, Figure S6G–I). The ROC curves of the TARGET-OS, GSE17674, and GSE63157 cohorts displayed the predictive ability of the prognostic model (Figure S6J–L); in particular, with a favorable discriminating power, it demonstrated that the OS of the 1-, 3-, and 5-year AUCs was 0.719, 0.648, and 0.590 in the TARGET-OS dataset.

### 2.7. Validation of the LMAGs Risk Model by the CHCAMS Cohort

**Figure 6:**
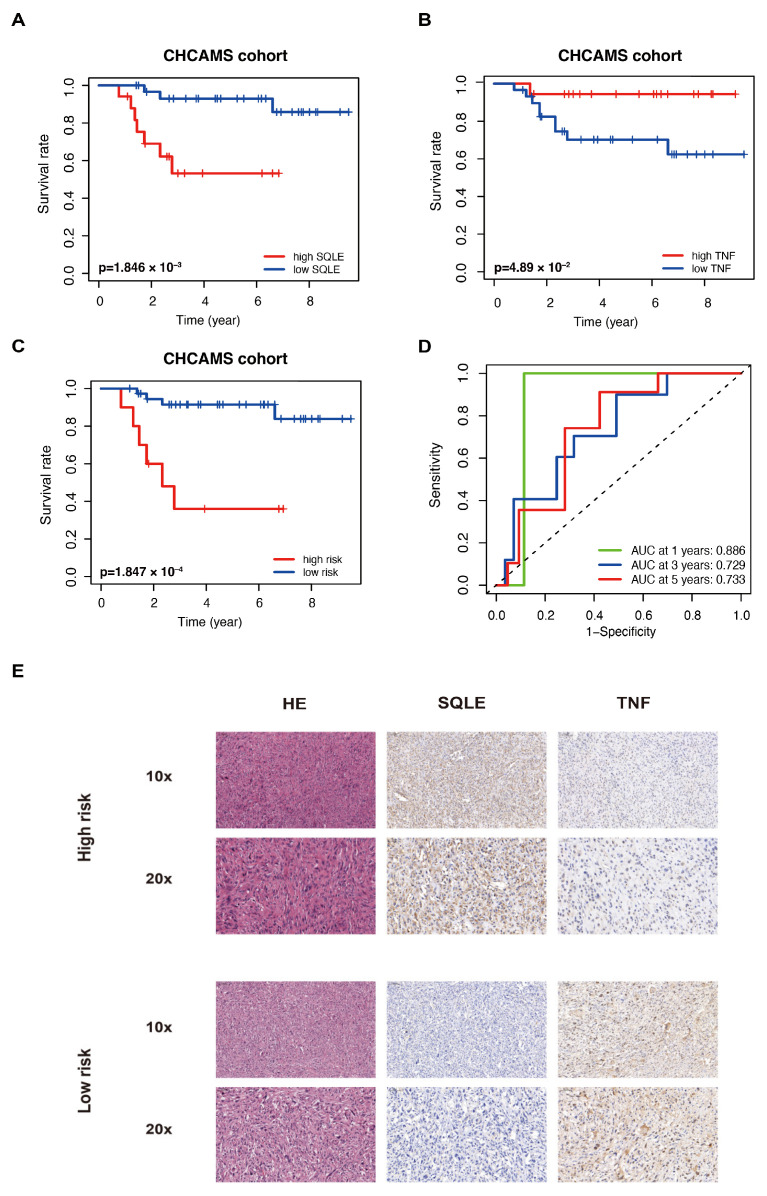
LMAGs risk model validation using independent CHCAMS cohorts. The SQLE (**A**) and TNF (**B**) prognostic value in the cohort. (**C**) OS in the CHCMAS cohort. (**D**) Analysis of the ROC. (**E**) Representative histological staining images of the cases in this cohort.

Forty-eight sarcoma samples were collected at CHCAMS to further validate the risk model. Table S6 shows the clinical characteristics of the patients. The individual expression of SQLE and TNF in sarcoma demonstrated opposite prognostic prediction (Figure 6A,B). Patients with sarcoma were divided into high-risk and low-risk groups based on their lipid metabolism-associated risk score. Using the same formula, we calculated the risk score as well. A lower OS was observed in the CHCAMS cohort of high-risk patients (Figure 6C), and at 1, 3, and 5 years, the AUCs were 0.886, 0.729, and 0.733, respectively (Figure 6D). Representative images of histological staining are shown in Figure 6E. 

### 2.8. SQLE as a Potential Target for Therapy

**Figure 7:**
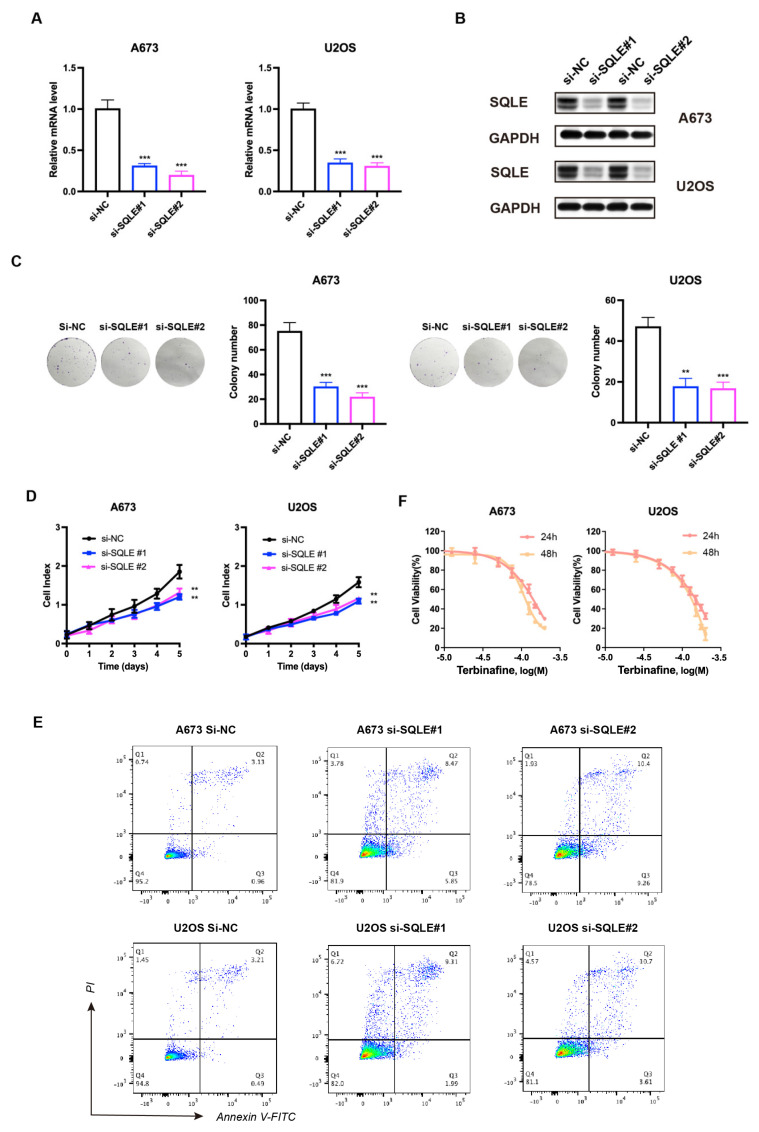
Knockdown of SQLE represses sarcoma cell proliferation in vitro. (**A**) mRNA and (**B**) protein levels of SQLE post–siRNA transfection. GAPDH was used as an internal control. (**C**) Colony formation and (**D**,**E**) cell proliferation post–siRNA transfection. (**F**) Cell viability was normalized by DMSO controls of A–673 and U2OS cells treated for 24 h and 48 h with respective Terbinafine concentrations. The results were acquired from triplicated experiments. ** *p* < 0.01; *** *p* < 0.001.

Cholesterol is known to be essential in membrane biogenesis and a precursor of steroids. In cholesterol biosynthesis, SQLE is a rate-limiting enzyme [18,19]. Several studies have revealed that SQLE is over-expressed in various tumors, acting as an oncogene [20,21,22]; however, its function in sarcoma remains uncertain. To investigate the contribution of SQLE to the prognostic model, we first knocked down SQLE in A-673 and U2OS cell lines. A qPCR and immunoblot analysis revealed that SQLE expression was reduced both at the mRNA and protein levels (Figure 7A,B). Furthermore, silencing SQLE substantially inhibited cell proliferation and colony formation but promoted the apoptosis of sarcoma cells (Figure 7C–E), confirming that SQLE is an oncogenic protein. Moreover, we found that terbinafine, a specific SQLE inhibitor, inhibited cell viability in a dose- and time-dependent manner (Figure 7F). According to these results, targeting SQLE could suppress proliferation and induce the apoptosis of sarcoma, implying that SQLE might be a therapy target.

## 3. Discussion

The molecular stratification of cancer aims to provide an accurate diagnosis of the disease and enhance the predictive ability of targeted therapies in terms of drug sensitivity [23]. The application of immunotherapy to improve the survival of sarcoma patients is a promising strategy with successive immunotherapy in tumor treatment [24,25]. Previous studies have well documented the obvious effects of immunotherapy based on immunostimulants and immune cells [26]. Early research concerning subclustering of sarcoma mainly focused on immune subgroups as well [27]. However, not all malignancies benefit from immunotherapy; for instance, clinical trials of immunotherapy for sarcomas showed fewer positive responses [28]. Hence, it is essential to stratify patients and find out potential drugs and therapies for clinical appliance.

This study was a comprehensive analysis of molecular stratification using both public datasets and data from patients in the CHCAMS cohort. We integrated information on gene expression profiles and survival outcomes and revealed the prognostic value of metabolic signature and the immune infiltration of high- and low-risk patients, thus proposing various therapeutic recommendations for sarcoma. High proportions of immune cells were observed in the TCGA-SARC samples from low-risk sarcoma patients, including CD8+ T cells, monocytes, M0 and M1 macrophages, and resting dendritic cells. Moreover, immune checkpoint gene expression was increased in these patients, suggesting that the risk score model can predict sarcoma patients’ response to immunotherapy.

As a precursor to steroids, cholesterol is essential in membrane biogenesis, and SQLE is an enzyme that limits the rate of cholesterol biosynthesis [18,19]. Studies demonstrated that SQLE was found to be over-expressed in many tumors, functioning in cell proliferation, epithelial-to-mesenchymal transition, and therapy resistance by activating certain pathways [20,21]. Targeting SQLE could promote the efficacy of radiotherapy in non-small cell lung cancer and breast cancer by interrupting homologous recombination via the endoplasmic reticulum (ER) stress response [29]. This paper indicated that therapeutics targeting SQLE could extend survival in patients with high-risk sarcoma. In our study, SQLE knock-down suppressed proliferation and induced the apoptosis of sarcoma cells, strengthening the notion that SQLE has a targetable oncogenic function in sarcoma.

Currently, terbinafine targeting SQLE is an essential anti-fungal drug clinically used for the treatment of superficial mycosis [30]. As for sarcoma, terbinafine has not been applied in clinical application yet. However, previous research indicates that terbinafine exhibits therapeutic potential for tumors; for instance, this drug regulates tumor progression and angiogenesis [20,31,32]. A study concerning colorectal cancer (CRC) revealed that terbinafine repressed tumor cell proliferation in vitro and in vivo [33]. A population-based study demonstrated that patients who received terbinafine post-diagnosis of CRC underwent a declined risk of death and metastasis (HR = 0.50; HR = 0.44) compared to those without treatment of terbinafine [34].

TNF, originally identified as a pleiotropic cytokine, mediates innate immunity which is capable of inducing hemorrhagic necrosis of tumors; therefore, the history of this factor is tightly correlated with tumor immunotherapy [35]. However, clinical trials using systemic TNF administration have caused unpredictable toxic effects that have impeded its development [36,37]. In contrast, localized TNF administration using isolated limb perfusion has yielded excellent effects in soft tissue sarcomas [38,39]. A pilot study reported a remarkable decrease in the number of CD4+ T-cells (*p* = 0.037) and the level of TNF (*p* = 0.004) in sarcoma patients [40]. It is known that tumor tissues are infiltrated with T cells, monocytes, and other cells producing TNF. The regulation of immune response in sarcoma patients was also determined through the measurement of several cytokines including TNF produced by Con-A-stimulated T cells. The level of TNF from Con-A-stimulated blood lymphocytes of sarcoma patients was found to be reduced compared to the controls. The enhanced level of SQLE and the reduced levels of TNF observed in sarcoma patients in this study indicate that the synergistic effects of both factors may support tumor angiogenesis and progression.

Apart from lipid metabolism, dysregulated signaling pathways have been reported to lead to the development of osteosarcoma. For instance, the Wnt pathway is involved in bone formation, and research on the transcriptomic analysis of osteosarcoma revealed that the Wnt pathway is inhibited and adiponectin, which is upregulated and related to the inhibition of the Wnt pathway, is a potential biomarker [41]. Also, the insulin receptor involves metabolic regulation and forms a hybrid receptor with IGF1R for IGF1, the latter together with IGF2, to promote the proliferation of sarcoma cells. The mRNA levels of IGF1 and IGF2 were enhanced, but the expression of IGF1R was unaltered in sarcoma, indicating that tumor proliferation might be promoted by an increased effect of IGF1 and IGF2 [42].

## 4. Materials and Methods

### 4.1. Data Acquisition

#### 4.1.1. Public Datasets

Transcriptome profiles and sample information for sarcoma were obtained from the Cancer Genome Atlas (TCGA; https://portal.gdc.cancer.gov/, accessed on 12 November 2022) and Gene Expression Omnibus (GEO; accession number: GSE63157 and GSE17674; https://www.ncbi.nlm.nih.gov/gds, accessed on 12 November 2022) databases. Osteosarcoma sample information was obtained from the Therapeutically Applicable Research to Generate Effective Treatments (TARGET; https://ocg.cancer.gov/programs/target, accessed on 12 November 2022) database. The TCGA patients were randomly divided into the training and test sets (1:1 ratio) (Table S1), and the cohorts from TARGET and GEO were treated as external validation datasets. A total of 584 genes related to lipid metabolism were downloaded from the AmiGO 2 Web portal (http://amigo.geneontology.org/amigo/landing, accessed on 12 November 2022) and the GeneCards (https://www.genecards.org/, accessed on 12 November 2022) and National Center for Biotechnology Information (NCBI, https://www.ncbi.nlm.nih.gov/, accessed on 12 November 2022) databases.

#### 4.1.2. CHCAMS Cohort

Our cohort enrolled sarcoma patients (N = 48) at Cancer Hospital, Chinese Academy of Medical Sciences (CHCAMS) from 2010 to 2022. Patients with complete clinical and follow-up data were included. Ethics Committee approval was obtained from CHCMAS for the study protocol. Written informed consent was provided by all patients.

### 4.2. Sarcoma Subcluters Identification 

The obtained lipid metabolism-associated genes (LMAGs) were then used in non-negative matrix factorization (NMF) clustering [43]. We excluded candidates with low median absolute deviation (MAD) values (MAD ≤ 0.5). Analysis of Cox regression was then performed to assess the association between LMAGs and OS using the “survival” R package, and to capture candidate LMAGs related to prognosis. Eventually, unsupervised NMF clustering was applied to the TCGA sarcoma data. To determine the k value, cophenetic correlation coefficients were calculated (k = 2 to 7, Figure S1). As a result, k = 2 was chosen as the best cluster number.

### 4.3. The Construction and Validation of LMAGs

The LASSO Cox regression analysis was used to screen LMAGs to minimize overfitting of the model using the “glmnet” R package [44]. A novel prognostic model was constructed using multivariate Cox proportional hazards regression analysis [45]. The risk score of each sample was calculated by the formula: risk score = (0.838946625 × SQLE) + (−2.492672995 × TNF). Each cohort was divided into low- and high-risk groups according to the median cut-off value of risk score. By using the R packages “survival” and “survminer”, we conducted an analysis of the optimal cut-off and the Kaplan–Meier survival curve to analyze the survival conditions of the signature. The receiver operating characteristic curve (ROC curve), risk plot, and concordance index (C-index) were used to determine the predictive ability [46]. A nomogram was developed based on clinical properties that are independent prognostic factors in the multivariate analysis [47]. To predict 1-, 3-, and 5-year survival, we used the scores associated with clinical properties. Calibration plots comparing predicted and observed death probabilities at 1, 3, and 5 years were used to assess model calibration.

### 4.4. Analysis of Tumor Microenvironment and Clinical Treatment Response

We evaluated the correlation between immune cells and risk features using several commonly used approaches, including TIMER, EPIC, QUANTISEQ, XCELL, CIBERSORT, MCPOUNTER, and CIBERSORT-ABS. To assess the correlation between immune infiltrates and risk scores, a Spearman’s correlation analysis was performed. Ultimately, we sought to determine whether the two groups differed in gene expression in immune checkpoints. Sarcoma mutation data were downloaded from the TCGA data portal (https://portal.gdc.cancer.gov/, accessed on 12 November 2022) and stored in MAF format. TMB (tumor mutational burden) was calculated using the R package “maftools” as mutations per million bases [48]. The “estimate” R package evaluated the STromal and Immune Cells in Malignant Tumors (ESTIMATE) score [49]. Using GDSC, the “oncoPredict” package in R was used to predict the IC50s of drugs [50].

## 5. Conclusions

In conclusion, in vivo experiments are required to further confirm the putative targets identified in our analysis. Targeting lipid metabolism and cholesterol biosynthesis deserves more robust therapeutic approaches for sarcoma. This study provides a comprehensive description and improves the understanding of sarcoma. The predictive models and SQLE might serve as valuable hints for the in-depth exploration of sarcoma diagnoses and therapies.

## Data Availability

Publicly available datasets were analyzed in this study. The data can be found at The Cancer Genome Atlas (TCGA) (https://portal.gdc.cancer.gov/, accessed on 12 November 2022); Gene Expression Omnibus (GEO) (https://www.ncbi.nlm.nih.gov/gds, accessed on 12 November 2022); the Therapeutically Applicable Research to Generate Effective Treatments (TARGET) (https://ocg.cancer.gov/programs/targethttps://www.ncbi.nlm.nih.gov/gds, accessed on 12 November 2022). The datasets generated during the current study are available from the corresponding author upon reasonable request.

## Supplementary Materials

The following supporting information can be downloaded at: https://www.mdpi.com/article/10.3390/ijms25031643/s1.
