# Supplementary material for: Transcriptome Sequencing Unveils a Molecular-Stratification-Predicting Prognosis of Sarcoma Associated with Lipid Metabolism
Source: Int J Mol Sci. 2024 Jan 29;25(3):1643. doi: 10.3390/ijms25031643 (PMC10855378; doi:10.3390/ijms25031643)
Supplement: Supplementary file 1 [file ijms-25-01643-s001.zip › Supplementary Materials Methods and Captions.pdf]

Supplementary materials for

Transcriptome Sequencing Unveils Lipid Metabolism-Associated Molecular Stratification Predicting Prognosis of Sarcoma

Materials and methods:

Histology staining

Tissue samples were firstly fixed in 10% formalin buffered with phosphate (pH 7.2–7.4), embedded in paraffin, subsequently, cut into 5 μm sections, stained with hematoxylin and eosin (HE), finally evaluated by light microscopy.

After deparaffinization, rehydration, and antigen retrieval, tissues were incubated with primary SQLE (1:300; Cat No.12544-1-AP; Proteintech, Wuhan, China) or TNF (1:400; Cat No.60291-1-Ig; Proteintech) antibodies and stained with 3,3′-diaminobenzidinetetra hydrochloride (DAB). The following criteria of staining was used to determine the intensity: 0—no, 1—weak, 2—moderate, and 3—strong. Staining scores were reviewed by two pathologists.

Cell and reagent

The human Ewing's sarcoma cell line A-673 and osteosarcoma cell line U2OS from the American Type Culture Collection (ATCC, VA, USA) were examined and authenticated by short tandem repeat (STR) profiling. The cells were mycoplasma negative and cultured in Dulbecco's modified Eagle medium (DMEM; Gibco, Grand Island, NY, USA) supplemented with 10% fetal bovine serum (FBS; Gibco) and 1% Penicillin/Streptomycin (P/S) (Gibco), at 37°C with an atmosphere of 5% CO2. Terbinafine was purchased from Selleckchem (Houston, TX, USA).

Gene silencing

Cells were cultured in 6-well plate to 60-70% confluence, and subsequently were transfected with 50 nmol NC and SQLE siRNAs using 5 μl Lipofectamine 3000 reagent (Invitrogen) separately. The targeted oligos were: 5′-CUGGAGAUAUCAAGGAACUTT (siSQLE#1); 5′-CCACUGACAAUUCUCAUCUTT (siSQLE#2); 5′-GCUCAGGCUCUUUAUGAAUUA (siSQLE#3).

Real–time PCR

Total RNA extracted from cells with Trizol (Takara, Dalian, China) was digested with DNase I (Takara) according to the manufacturer's instructions. The real-time PCR reactions were conducted in triplicate using the SYBR Green master mix (Takara). GAPDH was used as an internal control. The qPCR primer sequences were: SQLE, forward, 5′-GATGATGCAGCTATTTTCGAGGC; reverse, 5′-CCTGAGCAAGGATATTCACGACA. GAPDH, forward, 5′-CTTCACCACCATGGAGGAGGC; reverse, 5′-GGCATGGACTGTGGTCATGAG. QPCR was conducted after 48 h siRNA transfection.

Immunoblot

Protein was extracted with RIPA buffer (Thermo Fisher Scientific). The lysates were set

on ice for 30 min, and then centrifuged at 14,000 × g for 20 minutes at 4 °C. After BCA quantification, the supernatants were then mixed with 4× SDS buffer and subjected to SDS-polyacrylamide gel electrophoresis (SDS-PAGE). Sequentially, the immunoblots were probed with the SQLE primary antibody (Cat No. 12544-1-AP, Proteintech, Wuhan, China) and secondary antibody (Cat No. 7074, Cell signaling technology, Danvers, MA, USA). The levels of the target protein were normalized to GAPDH (Cat No. 5174, Cell signaling technology, Danvers, MA, USA) expression. Immunoblot was conducted after 48 h siRNA transfection.

CCK8 assays

The indicated siRNAs were introduced to cells within 12 hours after seeding in 96-well plates at a density of 4,000 cells per well. Additionally, different concentrations of Terbinafine were applied to the cells for 24 hours or 48 hours. CCK8 reagent (Dojindo, Kyushu Island, Japan) was added into the medium for measuremen at 450nm 2 h post incubation at 37°C according to the manufacture's recommendation.

Colony formation assays

Cells transfected by siRNAs were plated in 6-well plates at a density of 1,000 cells and cultured for two weeks. The cells were then fixed and stained with 0.1% crystal violet (Beyotime, Beijing, China).

Apoptosis analysis

Cell apoptosis was detected using the FITC Annexin V Apoptosis Detection Kit I (Cat No. 556547, BD Biosciences). Previously harvested cells were washed twice with ice-cold phosphate-buffered saline (PBS; Gibco) and subsequently re-suspended in 1×Binding Buffer at a concentration of 1×106/ml. Then, 100μl of the solution, 5μl of FITC Annexin V and 5μl PI were transferred to a culture tube followed by gentle vortex and incubation for 15 min at 25°C in the dark. Finally, 400 μl 1×Binding Buffer was added to each tube and flow cytometry was performed within 1h.

Statistical analysis

R software version 4.0.5 (https://www.r-project.org/), GraphPad Prism version 8.0.2 (GraphPad Software Inc., San Diego, CA, USA) and SPSS Statistics 23 (IBM Corp., Armonk, NY, USA) were used for statistical analyses. Survival was estimated according to the Kaplan-Meier method and log-rank test were performed. Statistical differences between categorical and continuous parameters were determined using X2 and Mann-Whitney tests, respectively. The data were shown as mean ± SD of at least 3 independent experiments. The comparison of indicated two groups was performed by Student's t-test (two-tailed, unpaired): *$p < 0.05$; **$p < 0.01$; ***$p < 0.001$; ns, not significant.

Supplementary Figure legends:

Figure S1. Unsupervised NMF clustering of TCGA-SARC. (**A**) The correlation of cophenetic, dispersion, evar, residuals, rss, silhouette and sparseness coefficients with respect to the number of clusters. (**B**) Heatmap of NMF clustering numbers ranging from 2 to 7.

Figure S2. Correlations of lipid metabolism-associated genes in TCGA-SARC.

Figure S3. Construction of the LMAGs risk model. (**A**) LASSO coefficient profiles of the LMRGs. (**B**) The selection of lambda in the LASSO model by 1000-fold cross-validation based on minimum criteria for OS. (**C**) Forrest plot showing the 2 genes in the final model generated by the TCGA training cohort. Correlations of OS and expression of (**D**) SQLE and (**E**) TNF in TCGA-SARC. Expressions of (**F**) SQLE and (**G**) TNF in the two groups in TCGA-SARC.

Figure S4. Prognostic value of LMAGs risk model in different subgroups in TCGA-SARC. OS of the two groups according to (**A**,**B**) age, (**C**,**D**) gender, (**E**,**F**) metastatic status, and (**G–I**) subtypes of the patients. UPS, undifferentiated pleomorphic sarcoma; DDL, dedifferentiated liposarcoma; LMS, leiomyosarcoma.

Figure S5. Immune infiltration analyses of validation cohorts. Expression of immune checkpoint genes in two groups in (**A**) TARGET-OS and (**B**) GSE63157 cohorts. * $p < 0.05$, ** $p < 0.01$, *** $p < 0.001$. Estimation of STromal and Immune cells in MAlignant Tumours using Expression data (ESTIMATE) analyses of (**C–F**) TARGET-OS and (**G–J**) GSE63157 cohorts.

Figure S6. Prognostic value of LMAGs risk model in different cohorts. The distributions of risk score and overall survival status in the (**A**) TARGET-OS, (**B**) GSE17674, and (**C**) GSE63157 cohorts. The heatmaps demonstrated the expression of SQLE and TNF in the (**D**) TARGET-OS, (**E**) GSE17674, and (**F**) GSE63157 cohorts. The Kaplan-Meier curve of OS in the high- and low-risk groups in the (**G**) TARGET-OS, (**H**) GSE17674, and (**I**) GSE63157 cohorts. The analyses of ROC in the (**J**) TARGET-OS, (**K**) GSE17674, and (**L**) GSE63157 cohorts. TARGET, Therapeutically Applicable Research to Generate Effective Treatments database; TARGET-OS, TARGET osteosarcoma dataset.
