# Supplementary figures and images for: Transcriptome Sequencing Unveils a Molecular-Stratification-Predicting Prognosis of Sarcoma Associated with Lipid Metabolism
Source: Int J Mol Sci. 2024 Jan 29;25(3):1643. doi: 10.3390/ijms25031643 (PMC10855378; doi:10.3390/ijms25031643)

A

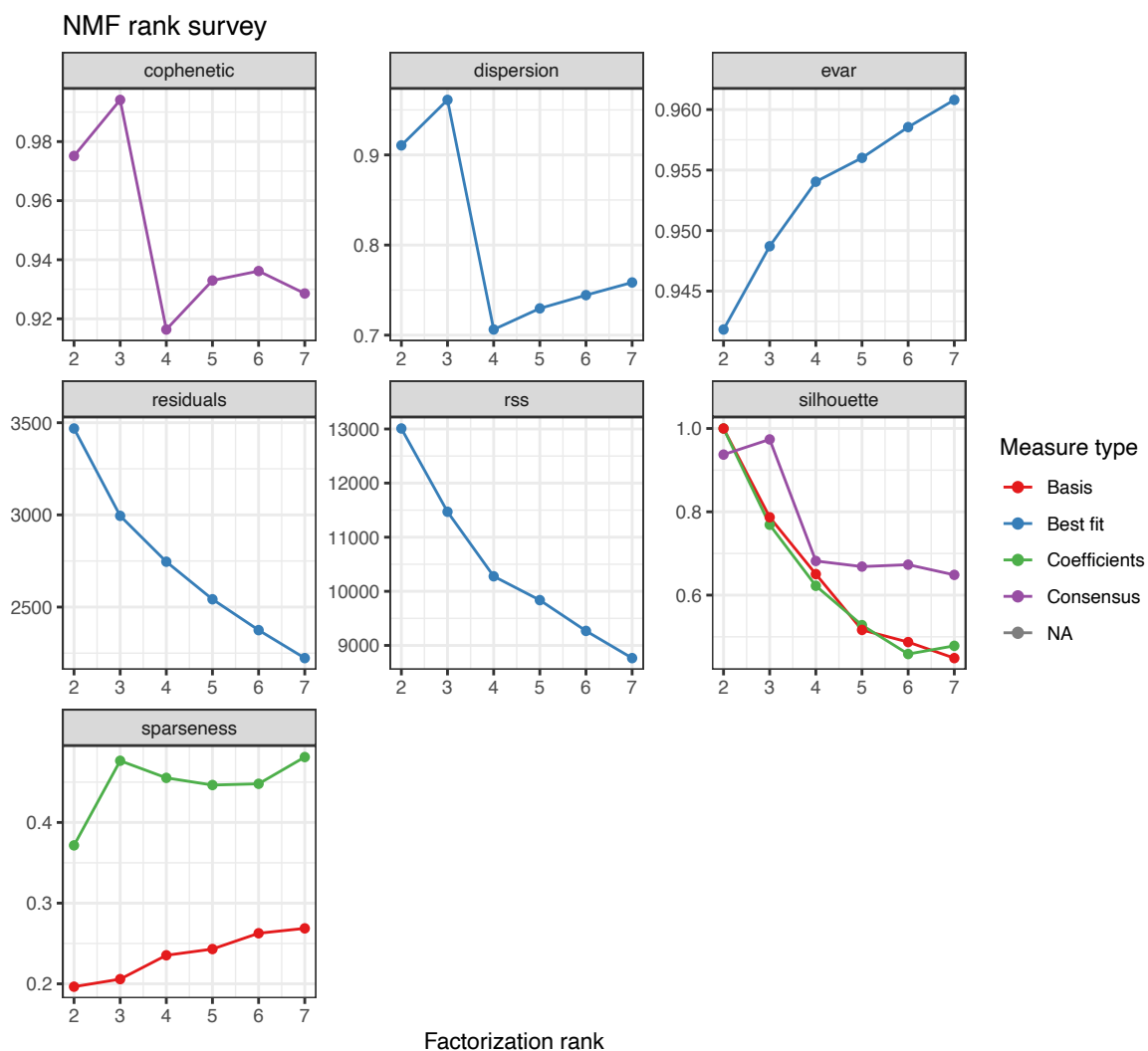

B

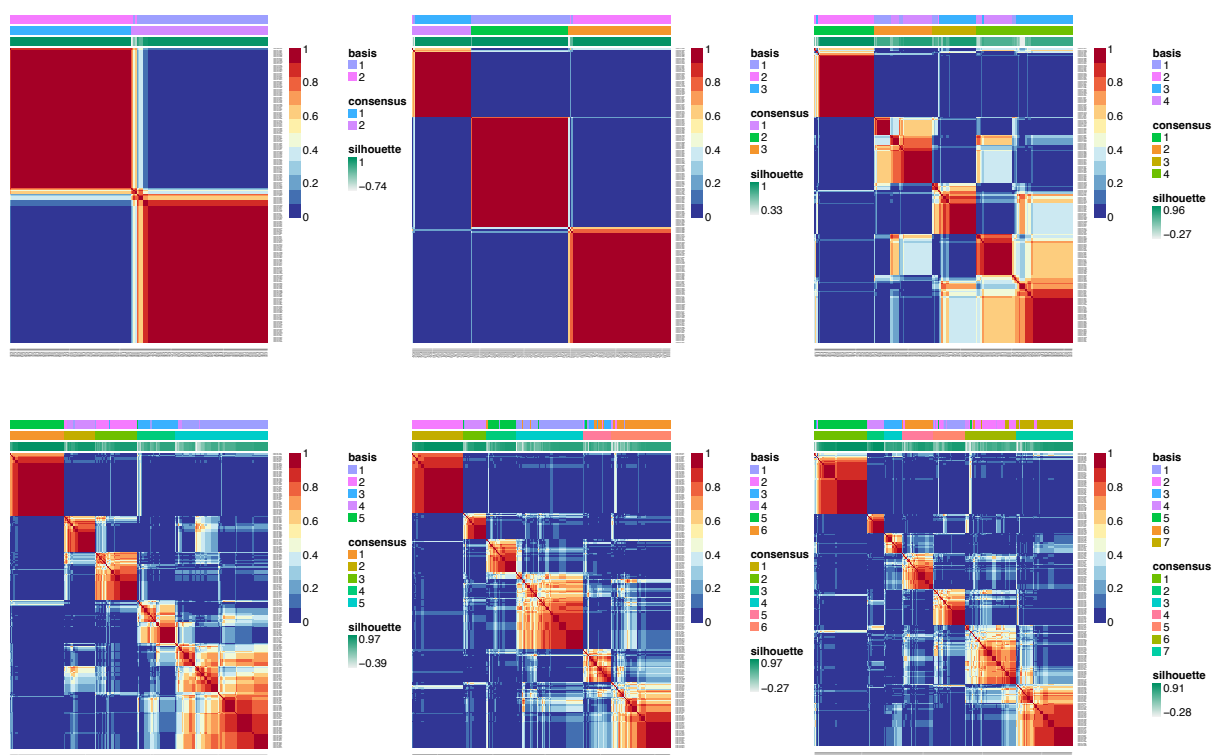

Supplement: Supplementary file 1 [file ijms-25-01643-s001.zip › Figure S1.pdf]

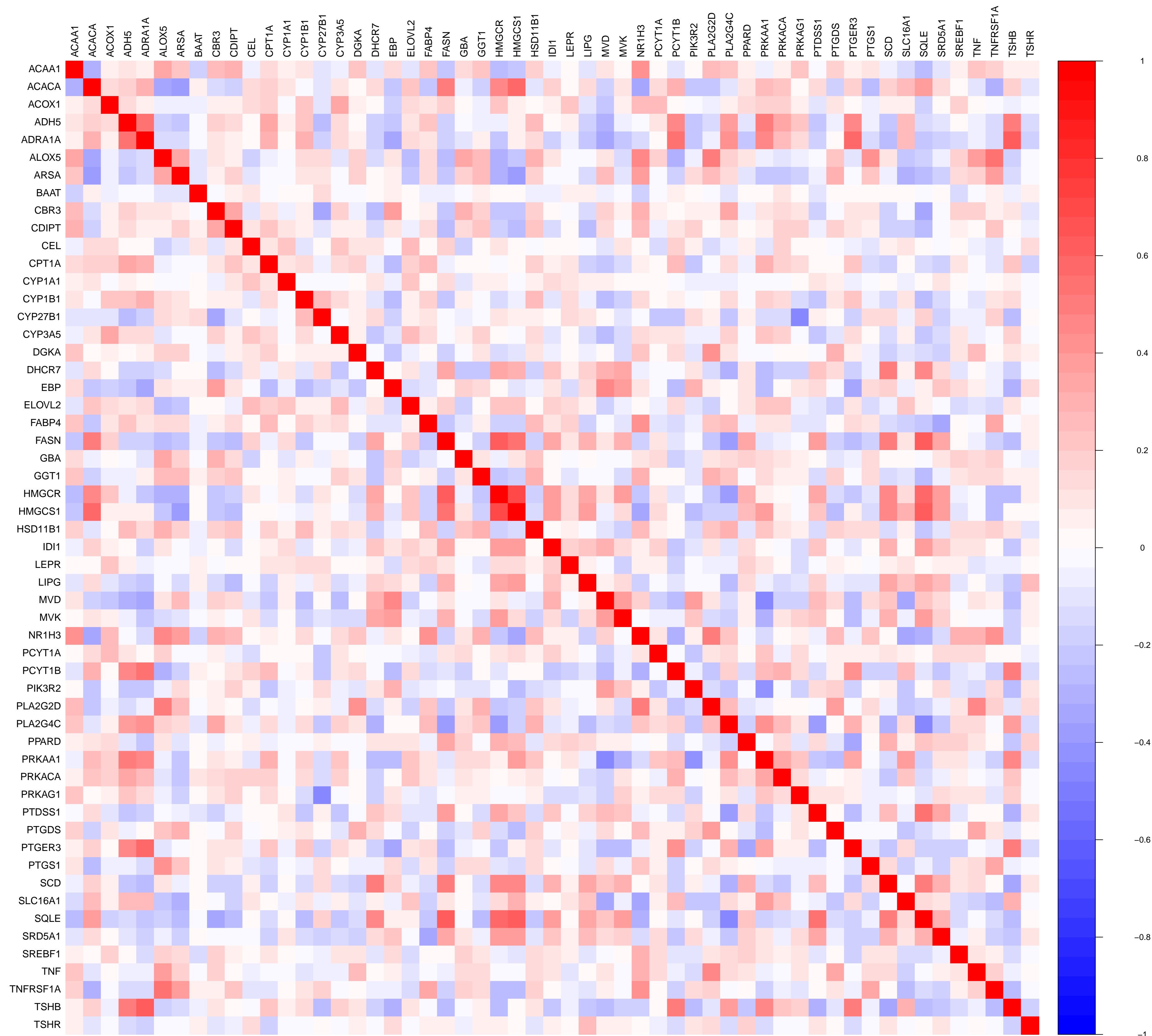

Supplement: Supplementary file 1 [file ijms-25-01643-s001.zip › Figure S2.pdf]

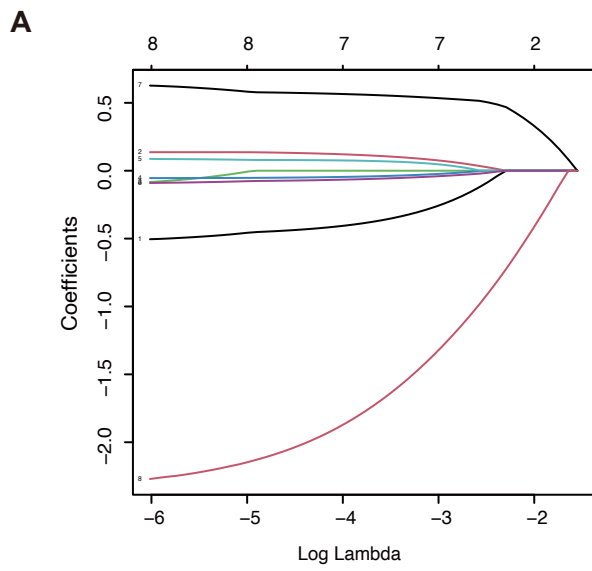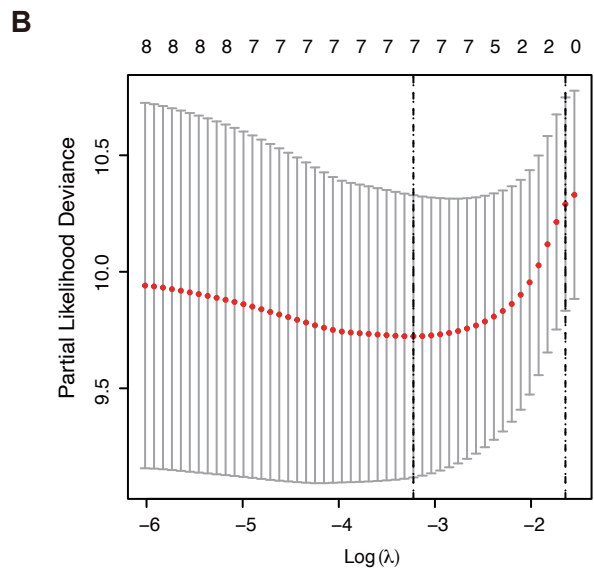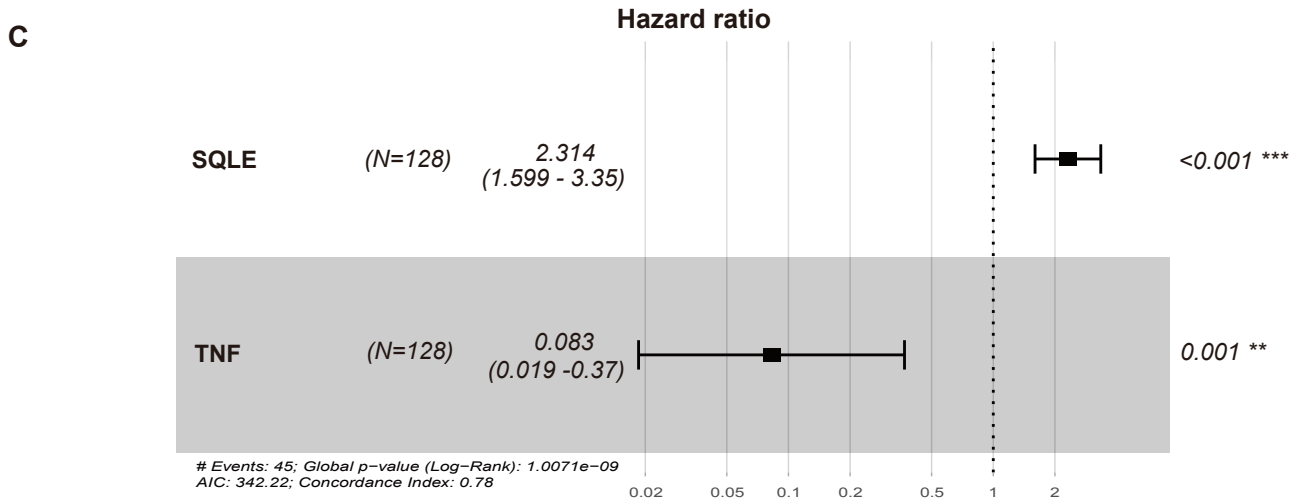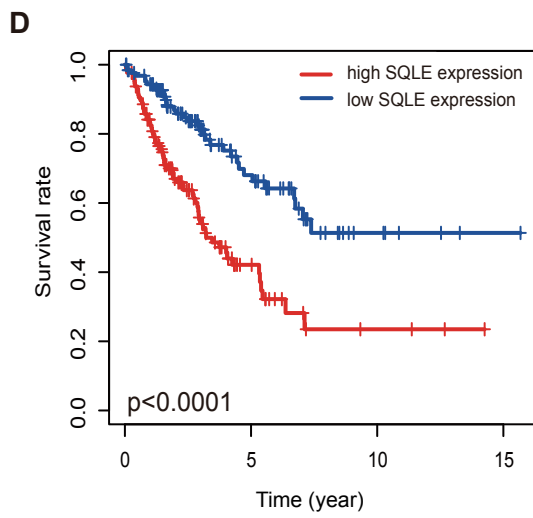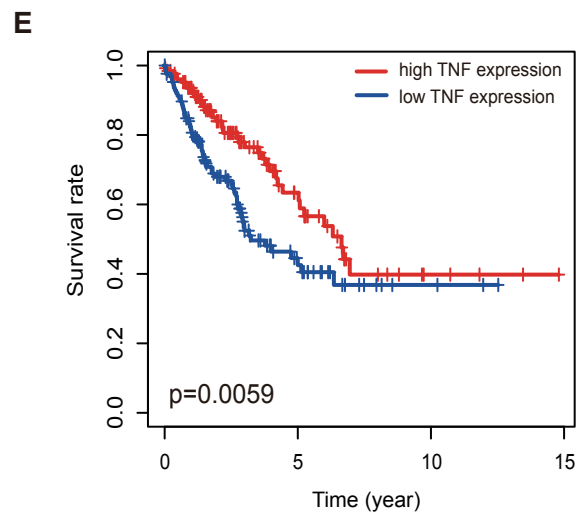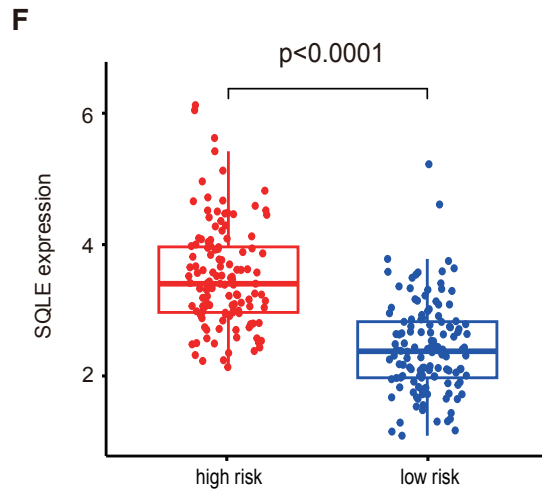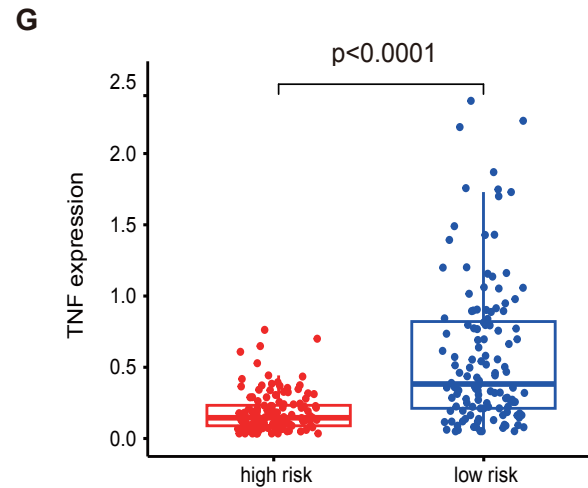

Supplement: Supplementary file 1 [file ijms-25-01643-s001.zip › Figure S3.pdf]

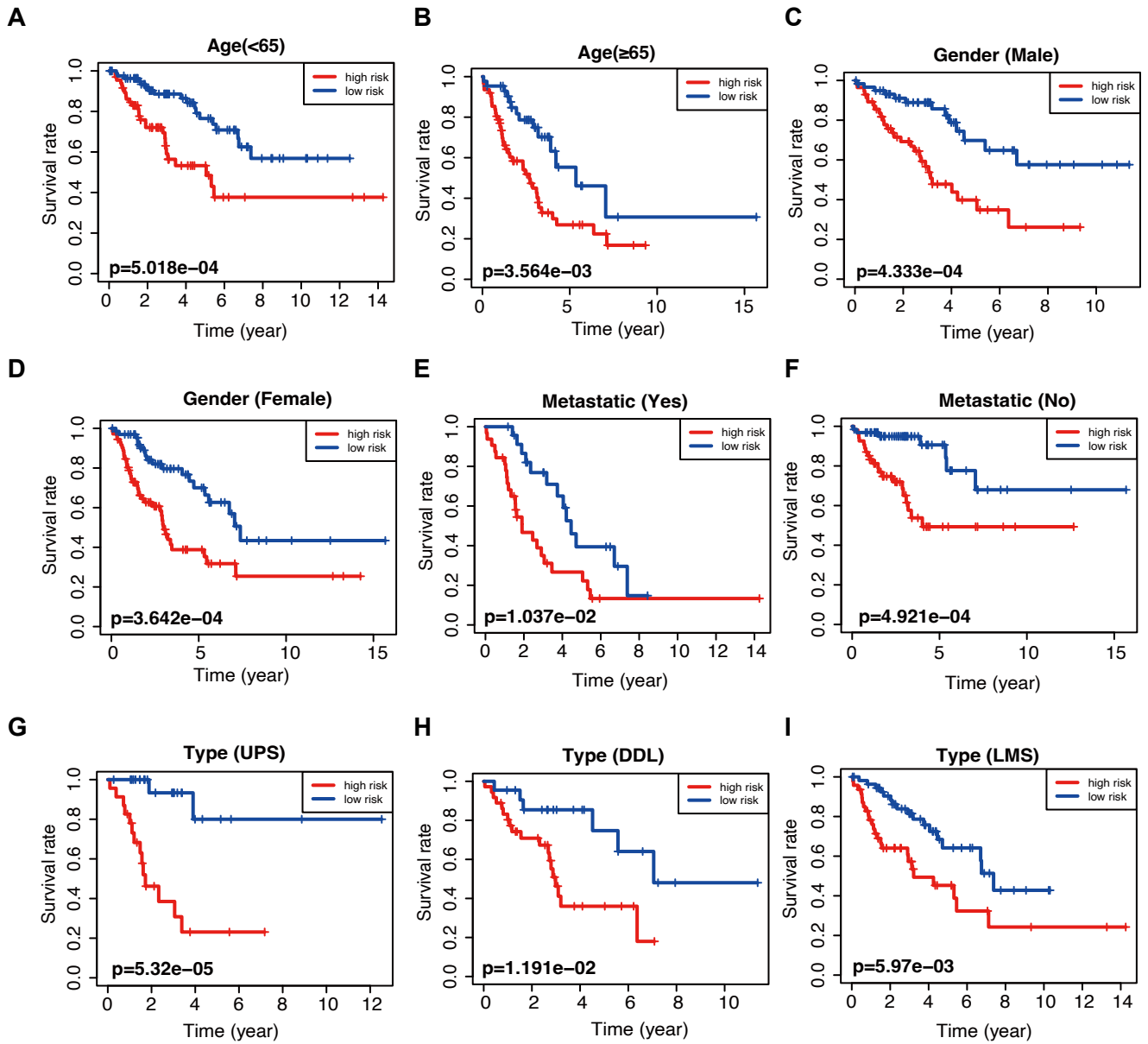

Supplement: Supplementary file 1 [file ijms-25-01643-s001.zip › Figure S4.pdf]

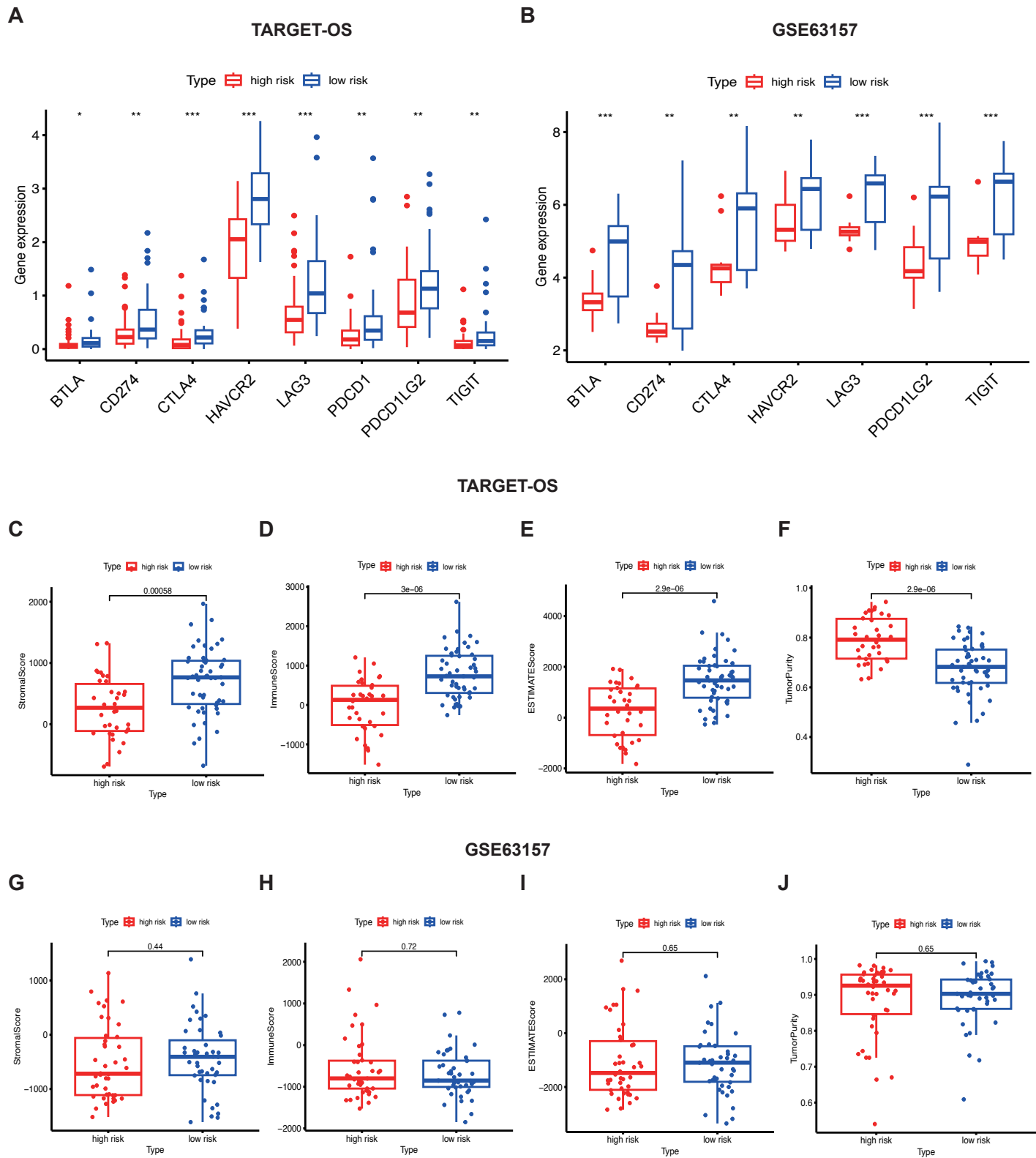

Supplement: Supplementary file 1 [file ijms-25-01643-s001.zip › Figure S5.pdf]

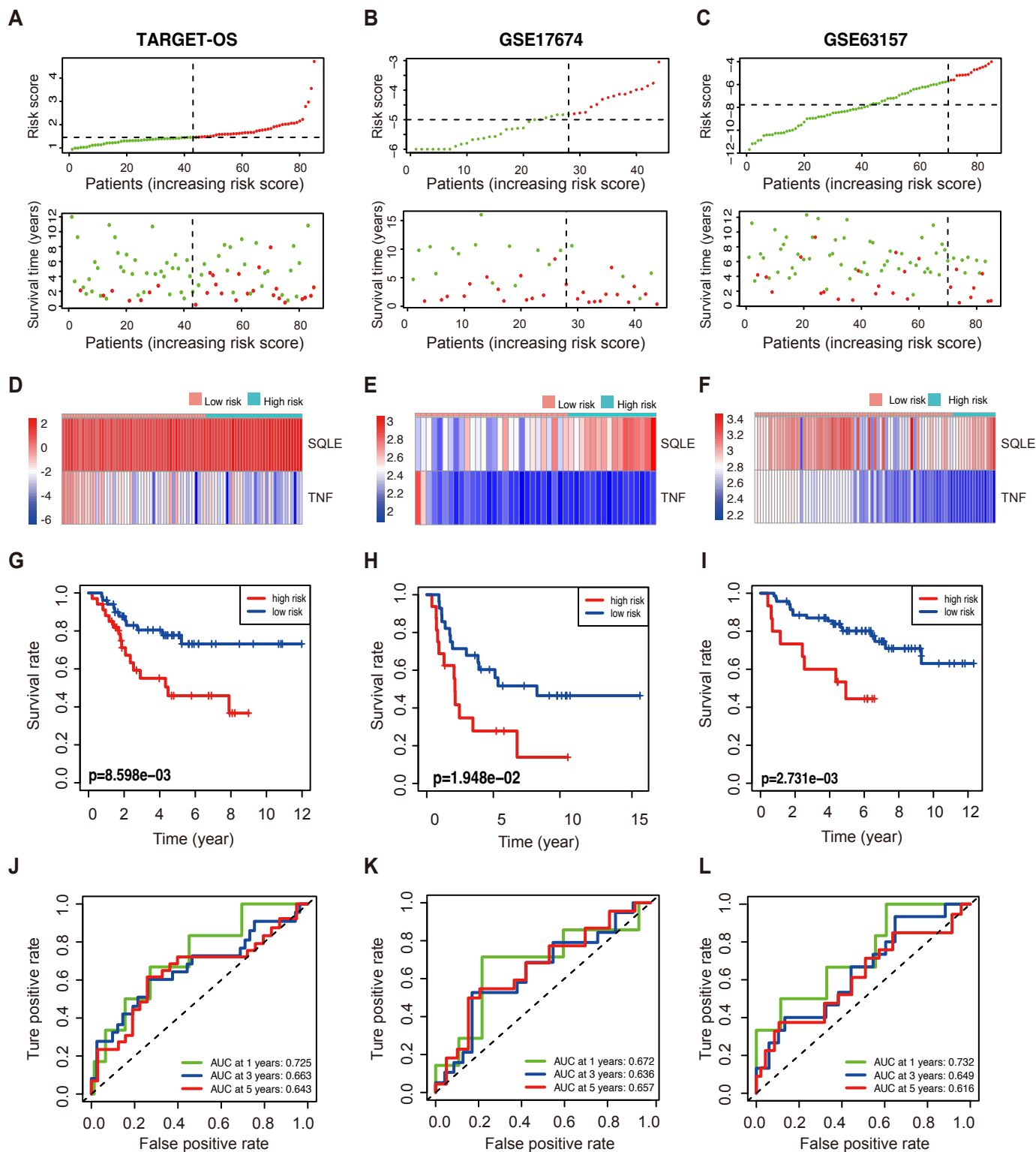

Supplement: Supplementary file 1 [file ijms-25-01643-s001.zip › Figure S6.pdf]
